# Complete genome sequence of a methicillin-resistant *Staphylococcus aureus* strain 63-2498, isolated from a diabetic foot infection

**DOI:** 10.1128/mra.00572-25

**Published:** 2025-09-19

**Authors:** Felise G. Adams, Sylvia A. Sapula, Bradley J. Hart, Morgyn S. Warner, Peter G. Speck

**Affiliations:** 1 Molecular Sciences and Technology, College of Science and Engineering, Flinders University 1065, Adelaide, Australia; 2 Health and Biomedical Innovation, UniSA Clinical and Health Sciences, University of South Australia 1067, Adelaide, Australia; 3 Microbiology & Infectious Diseases, SA Pathology 10107, Adelaide, South Australia, Australia; 4 Faculty of Health and Medical Sciences, University of Adelaide 1066, Adelaide, South Australia, Australia; Loyola University Chicago, Chicago, Illinois, USA

**Keywords:** bacterial genome, staphylococci, hybrid assembly

## Abstract

Here, we report the complete genome sequence of methicillin-resistant *Staphylococcus aureus* 63-2498, isolated from an outpatient with a diabetic foot infection. The 2.82 Mbp genome with 2,663 predicted coding sequences was assembled using a hybrid approach combining PacBio HiFi long-read and Illumina short-read sequencing, resulting in one chromosome and three plasmids.

## ANNOUNCEMENT

Diabetes-related foot infections are a common and costly complication of diabetes mellitus. Infections are largely polymicrobial with *Staphylococcus aureus* frequently isolated (1, 2). The complex etiology of the diabetic foot compromises treatment success, promoting multidrug resistance and lower-limb amputation incidence (3).

This announcement describes the complete genome of *S. aureus* 63-2498 generated from PacBio and Illumina sequences. 63-2498 was originally isolated in 2017 from an infected heel wound of a diabetic patient by SA Pathology (Adelaide, Australia). Culturing and taxonomic identification have been described previously (4). Since acquisition, 63-2498 has been stored at −150°C in 25% glycerol and selected for comprehensive genomic analysis due to demonstrated virulence in a diabetic murine wound model and methicillin resistance (4, 5).

For long-read sequencing, DNA was extracted using the Macherey-Nagel NucleoSpin Tissue kit following the manufacturer’s “hard-to-lyse bacteria” protocol. After quality control, DNA (1 µg) was sheared with g-TUBEs (Covaris), and libraries were prepared using SMRTbell prep kit 3.0 (PacBio). The final library was size selected by selectively binding to 3.3× v/v diluted AMPure PB beads and sequenced on a PacBio Revio (24 h runtime) by the Australian Genomic Research Facility (AGRF - Brisbane, Australia). HiFi reads were assembled using Hifiasm (v0.19.7) (6). Approximately 1.5 Gbp of sequence was generated, totaling ~530× coverage of the 2.82 Mbp genome (*N_50_*, 6,127 bp). For short-read sequencing, genomic DNA was isolated on a QIAsymphony SP using the QIAsymphony DSP Virus/Pathogen kit (Qiagen). Libraries were prepared using the Nextera XT DNA kit (Illumina Inc.) with modifications, using half volumes for input DNA, amplification reagents, and tagmentation. Libraries were cleaned using the AxyPrep MAG PCR Clean-Up Kit (Corning Inc.), pooled manually, and sequenced on a NextSeq 550 with the NextSeq 500/550 Mid Output kit v2.5 (300 cycles) (Illumina Inc.) by SA Pathology. Quality filtering and adapter trimming were achieved using PRINSEQ (v0.20.4) through TORMES (v1.3.0) (7), generating 1,659,012 paired-end 150 bp reads (~89× sequence coverage). The strain 63-2498 was cultured overnight at 37°C onto mannitol salt agar (Oxoid, Thermo Scientific), and colony biomass was used for each DNA extraction.

Long-read sequences were processed and converted to FASTQ files using PacBio pbtk (v3.1.1). PacBio and Illumina sequence assembly integration was achieved using Hybracter (v0.7.3) (8). Base quality and adapter trimming were ensured using Filtlong (v0.2.1) (https://github.com/rrwick/Filtlong) and Porechop (v0.5.0) (9), respectively. Flye (v2.9.6) was used for long-read sequence assembly (10), and Plassembler (v1.8.0) (utilizing unicycler) (11) was employed to recover plasmid sequences (12). Sequences were polished using Medaka (v1.10.0) (https://github.com/nanoporetech/medaka) and genome origin set to *dnaA* using Dnaapler (v1.2.0) (13). Polypolish (v0.6.0) and Pypolca (v0.3.0) refined Illumina read assemblies (14, 15). ALE (v20220503) determined genome assembly accuracy and completeness (16), generating four circularized contigs, representing one chromosome and three plasmids (~530× sequence coverage). Sequences were analyzed using TORMES (v1.3.0) (7) and NCBI Prokaryotic Genome Annotation Pipeline (v6.7) (17) and relevant features are summarized (Table 1). CheckM (v1.2.2) determined 98.62% completeness, 0.24% contamination, and 0.00% strain heterogeneity (18).

**TABLE 1:** Genetic characteristics and GenBank accession identifiers of *S. aureus* 63-2498

Genetic entity name and type	Total length (bp)	G + C content (%)	No. of CDS^*a*^	No. of rRNAs	No. of tRNAs	Plasmid replicon initiation protein type^*b*^	Topology	Virulence genes^*d*^	Antimicrobial resistance genes^*e*^	GenBank accession ID
63-2498, chromosome	2,778,331	32.81	2619	16	59	ND^*c*^	Circular	*adsA, aur, cap8A, cap8B, cap8C, cap8D, cap8E, cap8F, cap8G, cap8L, cap8M, cap8N, cap8O, cap8P, clfB, ebp, esaA, esaB, essA, essB, esxA, fnbA, geh, hlb, hld, hlgA, hlgB, hlgC, hly/hla, hysA, icaA, icaB, icaC, icaD, icaR, isdA, isdB, isdC, isdD, isdE, isdF, isdG, lip, map, sbi, sdrC, sdrD, seg, sei, sem, sen, seo, seu, srtB, sspA, sspB* and *sspC*	*mecA*, *blaZ*, *norA*, *tet(38)*, *lmrS*, *arlR, arlS*, *mepA, mepR* and *mgrA*	CP157001
p63-2498_1, plasmid	35,402	30.35	39	0	0	5a (Rep_3 family), 20 (RepA_N family)	Circular	0	0	CP157002
p63-2498_2, plasmid	3,011	28.99	3	0	*0*	21 (Rep_1 family)	Circular	0	0	CP157003
p63-2498_3, plasmid	2,473	30.77	2	0	0	10 (RepL family)	Circular	0	*ermC*	CP157004

^*a*^ CDS, coding DNA sequences (with protein).

^*b*^ Determined by PlasmidFinder 2.0 (19) via the Center of Genomic Epidemiology (https://cge.food.dtu.dk/services/PlasmidFinder/).

^*c*^ ND, not determined.

^*d*^ Virulence genes were identified by screening each genomic sequence against the Virulence Factors Data Base (VFDB) (20) via Abricate (v1.0.1) (https://github.com/tseemann/abricate) and VirulenceFinder (v2.0) against *S. aureus* via the Center of Genomic Epidemiology (VirulenceFinder 2.0).

^*e*^ Antimicrobial resistance genes were determined by screening each genomic sequence against the ResFinder database (21) and the comprehensive antibiotic resistance database (CARD) (22) as part of the TORMES pipeline (v1.3.0).

Multi-locus sequence typing (v2.19.0) (https://github.com/tseemann/mlst) categorized 63-2498 to Sequence Type 22 (ST22). Encoded virulence and/or antimicrobial resistance genes are listed (Table 1). Default settings were used unless otherwise specified.

## Data Availability

This genome project is available at GenBank under BioProject PRJNA1112560 and BioSample SAMN41430741 with the following accession numbers, 63-2498 (chromosome); CP157001, p63-2498_1; CP157002, p63-2498_2; CP157003 and, p63-2498_3; CP157004. Raw sequencing reads from the Illumina and PacBio sequencing runs are accessible from the Sequencing Read Archive (SRA) repository under the accession numbers SRX28303850 and SRX28412108, respectively. The version described in this paper is the first version.
